# How Can the Roma Deal with the Health and Social Crisis Generated by the COVID-19 Pandemic? Inequalities, Challenges, and Successful Actions in Catalonia (Spain)

**DOI:** 10.1007/s40615-023-01736-w

**Published:** 2023-08-03

**Authors:** Andrea Khalfaoui, Tania Garcia-Espinel, Fernando Macías-Aranda, Silvia Molina Roldán

**Affiliations:** 1 Moray House School of Education and Sport, University of Edinburgh, Edinburgh, UK; 2 Faculty of Education and Sport, University of Deusto, Bilbao, Spain; 3 Roma and Social Innovation Programme, Department of Social Rights, Government of Catalonia, Barcelona, Spain; 4 Department of Teaching and Learning and Educational Organization, University of Barcelona, Barcelona, Spain; 5 Department of Pedagogy, Universitat Rovira i Virgili, Tarragona, Spain

**Keywords:** Roma, COVID-19, Health inequalities, Social inequalities, Successful actions

## Abstract

The Roma is the most excluded non-migrant ethnic minority in Europe, facing prejudice, intolerance, discrimination, and social exclusion in their daily lives. This has led to a huge gap in several social domains between the Roma and non-Roma created for centuries. The COVID-19 pandemic has only increased the social and health inequalities that the Roma faced. In this context, it is important to identify actions that have been successful in mitigating the effects that the COVID-19 has had in increasing such inequalities. This paper presents the findings of a mixed-method study carried out in Catalonia (Spain) with the participation of more than 500 Roma, who reported their experience. The study results confirm the significant vulnerability and the negative impact of the COVID-19 pandemic on the Roma communities. Our research also highlights several successful actions developed by the Integrated Plan for the Roma of the Catalan Government, such as health literacy and adult education, as having a positive impact on the quality of life of many Roma during the health and social crisis generated by the COVID-19 pandemic. This paper suggests that the lessons learned from Catalonia could be transferred to other contexts across Europe and guide decision makers to promote the social inclusion and quality of life of the Roma, protecting Roma communities during current and future pandemics.

## Introduction

Despite living in Europe for more than 600 years, the Roma is the largest and the most excluded non-migrant ethnic minority in the continent. This is translated into a huge gap in several social domains between the Roma and non-Roma, such as in education (68% of the Roma leave education early), employment (only 43% of Roma are in paid employment), health (about a quarter of the Roma do not have national health insurance), and housing (facing ethnic residential segregation, precarious housing, and overcrowded households) [1]. The combination of these inequalities perpetuates an inter-generational cycle of social exclusion [2]. Particularly in Spain, data from 2018 showed that poverty and social exclusion affected more than 80% of the Roma population, unemployment rates reached 52%, and only 17% of the Roma had finished compulsory secondary education. Also, 39% of the Roma reported being discriminated against in the previous year, healthcare services being one of the contexts of discrimination [3].

In the field of health, the Roma suffer a higher prevalence of communicable and non-communicable diseases, as well as shorter life expectancies (up to 15 years lower in some European countries). These inequalities are closely linked to the high levels of poverty and social and educational exclusion, which act as social determinants of health [4, 5]. This situation is aggravated by anti-Roma prejudices and institutional arrangements in healthcare systems that are not sensitive to minority cultures [6]. To overcome this situation, research identifies key aspects such as a higher presence of the Roma in the healthcare system [6], improving health communication [7], and enhancing cultural sensitiveness [2, 8].

The COVID-19 pandemic has especially affected the most vulnerable collectives, increasing previous inequalities in the social and health domains [9–11]. With regard to the Roma [12], it resulted in a much higher risk of death from COVID-19 [13], while antigypsyism increased [14]. Indeed, according to a study conducted in Spain during the COVID-19 lockdown, the health self-perception of the Roma worsened, with a relevant negative impact on their mental health; this was accompanied by a substantial reduction of economic income in more than half of the surveyed households, increased difficulties to accede to basic food, and an increase of discrimination [15].

Thus, it is important to identify successful actions to mitigate the inequalities and discrimination that COVID-19 increased and enhance well-being. Scientific literature evidences the importance of community-health partnerships between minority groups’ organizations and healthcare professionals [16], as well as local community support actions for vulnerable groups [10] and improved communication channels to ensure understanding of the situation of individuals and families [17]. Evidence-based actions that enhance communication and community involvement have been successful to counteract the exclusion of the Roma community [18–20] and other vulnerable groups during the COVID-19 pandemic [21–23].

Specifically, research has identified health literacy as a powerful predictor of health status [24], as higher health literacy levels have been associated with better health outcomes such as a lower probability of having a limiting illness or a lower probability of needing hospital visits [25]. Interventions addressed to improving the population’s health literacy have been effective in enhancing health outcomes, especially for disadvantaged populations who are especially affected by low health literacy levels, and thus it is considered a key strategy to address social inequality [25–27]. Particularly, health literacy programs are crucial to improve health literacy among the Roma [28]. In the context of the pandemic, health literacy has facilitated knowledge and access to information related to COVID-19 [29] and has been related to higher vaccination prevalence [30].

## Context of the Study

Our study was conducted in Catalonia (Spain). In the Spanish context, the Roma are full-fledged citizens as part of the population of Spain, and recognized as an ethnic minority by official institutions. From a formal standpoint, they have full voting rights, access to healthcare, housing, employment, education, etc. Their language of communication is Spanish or the languages of the different regions that make up the country (e.g. Catalan in Catalonia), just like any other citizen. Within the family context, they may communicate in a dialect of the Romani language, called *Caló*, but it is not widely used. The inequality faced by the Roma community lies in the real access to resources and services—which are legally recognized—and in the historical discrimination and antigypsyism they suffer, both socially and institutionally.

Within this context, this study focuses on the actions implemented by the Integrated Plan for the Roma of the Catalan Government (IPRCG) (Catalonia, Spain) to help the Roma community cope with the health and social crisis generated by the COVID-19 pandemic and prevent further inequalities. The IPRCG is a set of public policies implemented since 2005 to improve the situation of the Roma community in Catalonia. The IPRCG is rooted in scientific research and the participation of the Roma in the design, implementation, and development of the actions. The IPRCG has already demonstrated to contribute to the inclusion of the Roma, for instance in the field of education [31]. Thus, this study analyzes how it contributed to addressing the health and social inequalities faced by the Roma during COVID-19.

## Materials and Methods

The study responds to a twofold objective: (1) to analyze the situation of the Roma at the beginning of the COVID-19 pandemic in Catalonia, in terms of their living conditions (housing, employment, and social welfare services) and their health status regarding COVID infection; and (2) to analyze the impact that the IPRCG actions had in mitigating the negative consequences of the pandemic. For this purpose, a mixed-methods study was conducted with more than 500 Roma, who reported their experience through quantitative and qualitative data collection techniques.

### Quantitative Data Collection and Analysis

First, a questionnaire was used to gather information about the Roma situation at the beginning of the COVID-19 lockdown. The questionnaire was distributed 14 days after the state of alarm and the confinement was decreed in Spain, and it was open for data collection for 13 days (from March 27 to April 8, 2020). A total of 14 questions were asked about (a) demographic data, (b) SARS-COV-2 infection of participants and relatives, (c) housing conditions, (d) employment situation, and (e) knowledge of available aids to cope with COVID-19.

The questionnaire was built on an online platform, and it was sent via email to associations and via WhatsApp to individual contacts of the IPRCG, who in turn forwarded it to other contacts, following the snowball sampling strategy to achieve a wide distribution of the questionnaire. The final sample of the study was 493 participants (see Table 1). Considering an estimation of a total Roma population in Catalonia of 100,000 people,^1^this is a representative sample size with a confidence level of 95% and a margin of error of 4.405.

**Table 1:** Questionnaire sample

		*n*	%
Gender	Women	287	58.22%
	Men	206	41.78%
	TOTAL	493	100%
Age range	16–24	95	19.71%
	25–44	307	63.69%
	45 +	80	16.6%
	TOTAL^a^	482	100%

^a^There were 11 non-valid responses

For the data analysis, descriptive statistics were used to obtain a picture of the situation of the Roma during COVID-19 and identify potential vulnerabilities.

### Qualitative Data Collection and Analysis

Subsequently, 10 in-depth communicative interviews and 2 focus groups were conducted with participants of the actions that the IPRCG implemented during the COVID-19 pandemic (see Table 2). The participants are a representative sample of Romani citizens in Catalonia and Spain. All participants were full-fledged citizens with full rights to have access to community services (e.g. healthcare or education); however, they suffer educational, social, and labour inequalities, live in vulnerable neighbourhoods, and, for the most of them, face discrimination in several areas of their lives, sharing in this regard common elements with the Roma throughout Europe [32, 33].

**Table 2:** Participants’ profiles

Participant pseudonym	Gender	Actions of the IPRCG in which they participated			Profile description	Technique	
		Health literacy	Adult education	Overcome the digital divide and access to information		Interview	Focus group
John	M	X	X	X	Member of the IPRCG. Former AUG student. Current coordinator of the AUG and the preparation course to obtain CSEC	X	
Joyce	F	X	X	X	Cultural mediator in a city council	X	
Sylvia	F	X	X	X	Dynamizer of the IPRCG and AUG student. Dynamizer of the preparation course to obtain CSEC	X	
Christian	M		X		Dynamizer of the IPRCG and AUG former student. Dynamizer of the AUG	X	
Robert	M		X		AUG student	X	X
Lukas	M		X		Dynamizer of IPRCG and AUG former student	X	
Daniel	M	X	X	X	Worker in a social organization and GAU former student	X	
Victor	M	X	X	X	Member of the IPRCG and AUG former student	X	X
Luis	M	X	X	X	Member of the IPRCG and AUG former student	X	
Laura	F	X		X	Recipient of support by the IPRCG with subsidies paperwork	X	
Michael	M	X	X		Technician in a Roma organization and AUG former student		X
Sandra	F	X			Cultural mediator in a children’s service at the Catalan Government		X
Ana	F	X	X		Beneficiary of the educational actions developed by the IPRCG		X
Maria	F	X	X		Member of the IPRCG and former beneficiary of the educational actions developed by the IPRCG		X
Julia	F	X	X		AUG former student		X
Sara	F	X	X		Cultural mediator in a city council and AUG student		X

At the moment of the data collection, and thanks to the actions of the IPRCG, the participants in the interviews and focus groups were engaged either in university education or in adult education programs. Before having access to adult education offered by the IPRCG, 95% of them had not completed compulsory education. Participants reported their close relatives to suffer inequalities in the access to technologies during the confinement. As for the participants themselves, they had overcome the digital divide in their households due to their participation in educational activities framed in the IPRCG.

The actions implemented by the IPRCG in which the sample participated were:

1. *Health literacy.* Audio-visual materials were created and disseminated among the Roma about COVID-19 transmission, protective measures, and strategies to deal with confinement at home.
2. *Adult education*. Three free activities (educational courses) were offered: (a) educational programs to prepare Roma adults for the exam to obtain the *Compulsory Secondary Education Certificate* (*CSEC*) for people over the age of 18; (b) *Access to University Group* (*AUG*), to prepare Roma adults for the official university entrance exams for people over the age of 25 and 45; (c) educational course on *Mediation* to obtain an officially recognized degree by the University of Girona (Catalonia, Spain).
3. *Actions to overcome the digital divide and have access to information.* Several actions were conducted: (a) *Courses delivery through online platforms:* buying Zoom licenses, creation of virtual campuses, and regular contact via WhatsApp; (b) *Families’ access to electronic devices and other technology resources to do homework with their children*, which were facilitated via some associations; (c) *Support with administrative procedures*, such as aid applications for self-employed, minimum income guarantee applications, and administrative formalities related to healthcare or education, among others.

Both the interviews and the focus groups were conducted after the most critical situation of sanitary and social emergency finished, intending to obtain a retrospective evaluation of the impact that the actions of the IPRCG had. First, the individual interviews were conducted, and afterwards, the focus groups were organized, one with 3 men and one with 5 women, to deepen the issues that emerged in the interviews.

The qualitative data collection was conducted based on the communicative methodology, which focuses on identifying the transformative components of social reality that help overcome inequalities. It is characterized by facilitating an egalitarian dialogue between researchers and end-users of the research where both the exclusionary and transformative components of reality can be identified intersubjectively. The communicative methodology has been successfully used with Roma people to identify actions with social impact to overcome the inequalities they face [34]. During the COVID-19 pandemic, the communicative methodology has also been successfully used to overcome difficulties to conduct research with vulnerable groups [35].

For the data analysis, four categories of analysis were created, covering (a) impact on health, (b) impact on education, (c) impact on employment, and (d) other impacts.

The data collection was entirely conducted in Spanish, as all participants knew the language. The data collection followed all ethical standards for research involving human participants included in the Declaration of Helsinki. All the participants took part freely and were informed beforehand of the objectives, procedures, and implications of the study. Informed consents were collected electronically using an online form that was written both in Spanish and Catalan (co-official languages in Catalonia). The study was fully approved by the Ethics Board of the Community of Researchers on Excellence for All (CREA) (approval number 20230125).

## Results

### The Situation of the Roma in Catalonia at the Beginning of the COVID-19 Confinement: Health and Social Risks

In this section, we present the analysis of the data collected through the online questionnaire at the beginning of the pandemic, following the areas of vulnerability covered by the questionnaire.

#### a) Health: the situation of SARS-COV-2 infection of participants and relatives

At the moment of responding to the questionnaire, 0.41% of the sample had been infected (and confirmed by test) by SARS-COV-2, and 6.9% had not been tested but reported compatible symptoms. Data on confirmed cases of infection in Catalonia showed 7362 cases (0.10% of the population) in March 2020 and reached 26,557 (0.34% of the population) in April 2020.^2^ Therefore, the Roma had an infection rate higher than the overall population already at the beginning of the pandemic, according to our sample.

On the other hand, 25.15% of the sample reported having infected relatives, and an additional 8.5% reported having relatives with symptoms, thus 1/3 of the sample had relatives with COVID-19 or related symptoms (see Table 3). 18.26% reported having two or more relatives infected.

**Table 3:** Rates of SARS-COV-2 infection

		*n*	%
Respondents with COVID-19	Infected (confirmed with test)	2	0.41%
	Probably infected (with symptoms)	34	6.90%
	Not infected (confirmed with test)	20	4.06%
	Probably not infected (no symptoms)	437	88.64%
Respondents’ relatives with COVID-19	With relatives infected	124	25.15%
	With relatives probably infected (with symptoms)	42	8.52%
	Without relatives probably infected (no symptoms)	327	66.33%

#### b) Housing conditions during the confinement

The housing conditions explored with the questionnaire covered the number of people in the household, the number of minors in the household, and the size of the dwelling. Data shows that almost 40% of the respondents lived in homes of less than 70m^2^, 2/3 of the respondents (64.5%) lived in a household where 4 or more people were confined together, and one quarter (25.56%) of the households had 3 or more minors confined together (see Table 4).

**Table 4:** Housing conditions during the confinement

Home size			People confined together			Minors confined together		
	*n*	%		*n*	%		*n*	%
< 40m^2^	20	4.21%	1 person	11	2.23%	0 minors	117	23.73%
40–59 m^2^	52	10.95%	2 persons	56	11.36%	1 minor	119	24.14%
60–69 m^2^	116	24.42%	3 persons	91	18.46%	2 minors	114	23.12%
70–79 m^2^	131	27.58%	4 persons	121	24.54%	3 minors	96	19.47%
80–89 m^2^	64	13.47%	5 persons	116	23.53%	4 minors	19	3.85%
90–99 m^2^	36	7.58%	6 or more persons	81	16.43%	5 minors	6	1.22%
100–110 m^2^	19	4.00%				6 or more minors	5	1.01%
110–120 m^2^	5	1.05%						
120–140 m^2^	8	1.68%						
140–160 m^2^	18	3.79%						
> 160 m^2^	6	1.26%						

Of those living in homes of less than 60m^2^, almost 60% lived in households with 4 or more people confined together, and almost 35% lived in households with 5 or more people confined together. Eighteen percent of the households of less than 60m^2^ had 3 minors confined together (see Table 5).

**Table 5:** People confined together in homes of less than 60m^2^

People confined together in homes < 60m^2^			Minors confined together in homes < 60m^2^		
	*n*	%		*n*	%
1 person	3	4.17%	0 minors	19	26.39%
2 persons	12	16.67%	1 minor	21	29.17%
3 persons	14	19.44%	2 minors	17	23.61%
4 persons	18	25.00%	3 minors	13	18.06%
5 persons	17	23.61%	4 minors	2	2.78%
6 or more persons	8	11.11%	5 minors	0	0.00%
			6 or more minors	0	0.00%

#### c) Employment situation at the beginning of the pandemic

The employment status of the respondents reflected a difficult situation already at the beginning of the pandemic. 56.73% of the sample were unemployed; of those, 27.35% received some unemployment or welfare benefit. Additionally, 11.22% stopped receiving an income because they were self-employed, and more than 13% were affected by a temporary employment regulation order.^3^ Only 18.78% were working because they worked in an essential sector or because they could telework (see Table 6).

**Table 6:** Employment situation at the beginning of the pandemic

Employment status	*n*	%
Not working and not receiving any income	137	27.96%
Not working, but receiving some income (minimum income, non-contributory pension, disability pension, etc.)	83	16.94%
Affected by a temporary employment regulation order (ERTE)	64	13.06%
Stopped receiving income because they were self-employed	55	11.22%
Teleworking and receiving the salary normally	53	10.82%
Receiving an unemployment benefit	51	10.41%
Woking employed by a company because their sector remains open (supermarket, pharmacy, transport, etc.)	33	6.73%
Work leave (maternity, illness, etc.)	24	4.90%
Dismissed	7	1.43%
Working self-employed because their sector remains open	6	1.22%

#### d) Knowledge and use of available aids to cope with COVID-19

Regarding the special aids promoted to facilitate the population to cope with COVID-19, 87.42% were knowledgeable of at least one of the aids asked and 33% had applied for some of the aids. The most known aids were meal grants (60%) and the prohibition to cut water, electricity, or gas to vulnerable groups during the crisis (44.42%) (see Table 7).

**Table 7:** Knowledge and use of special aids to facilitate the population to cope with COVID-19

Knowledge of the aids	*n*	%
Knowledge of at least one aid	431	87.42%
Aid for self-employed	158	32.05%
Meal grants	296	60.04%
Postponing mortgage payments for affected workers	148	30.02%
Prohibition to cut the water, electricity or gas to vulnerable groups during the crisis	219	44.42%
Not knowing any aid	62	12.58%
Use of the aids	*n*	**%**
Have applied for some of the aids	163	33.06%

### Impact of the Actions of the Integrated Plan for the Roma to Alleviate the Consequences of the COVID-19

Results from the interviews and focus groups show that the actions developed by the IPRCG helped the participants to navigate better and with more confidence the lockdown situation in the Spanish context (March–May 2020). All the participants shared experiences on how these actions had positive impacts in the fields of health, education, and employment. The following sections delve into their experiences regarding those important components of society.

#### a) Impact on health: protecting from infection and protecting mental health

The explanatory videos facilitated by the IPRCG team were important for the participants to get a clear idea about the virus and learn effective measures to protect themselves from the spread; for instance, how to sanitize their hands and the proper use of the facemasks. Although the evidence about the virus and the measures to stop de spread were scarce, the IPRCG made sure that the existing evidence was available to everyone, especially the most vulnerable Roma households. John said: “Thanks to the scientific information they [IPRCG] shared, we realized that just following the official measures to stop the spread, we could overcome the pandemic all together” (John, Interview).

In this regard, according to the participants, it was important that the videos were made by Roma people and used an accessible style of language that made the information easily understandable, as Robert said: “Listening to it from Matias, from Lucia, and [Roma] people like them, I think that made us understand it better because they explained it with words that we understand better, without so many technicisms. Then, it gave us confidence”. (Robert, Focus group).

According to the experiences of the participants, the health information shared through the IPRCG was crucial in helping not only individuals, but entire Roma households navigate the pandemic. Indeed, as Christian stated, thanks to that information he was able to suggest his grandfather come to his house so that he could combat the situation together and not by himself. As an elderly and illiterate man, Christian’s grandfather had a great risk of being infected:

My grandfather was at my house during the lockdown because he did not go to school and never studied so he was very vulnerable to hoaxes that were spreading at that time. So, the health information we have had improved his health condition. (Christian, Interview)

All that information effort had positive consequences for the health status of many Roma families. Indeed, entire households, such as Lukas’ or John’s, never had the virus, as they explained in the interviews. This breaks with the prejudice, which circulated for months, that the Roma were guilty of spreading the virus because they usually live in crowded houses.

Along with this, IPRCG actions also had a positive impact on end-users’ mental health. According to Victor, the support and scientific information shared by the IPRCG generated a network that alleviated the loneliness and anxiety that many citizens experienced during the first stages of the pandemic. As Victor recalls: “The IPRCG was an important escape mechanism from the frustration caused by the isolation during the lockdown” (Victor, Interview).

Laura insists on this idea when she recalls her experience during the lockdown. She was active in participating in online educational activities of the IPRCG and her implication gave her a purpose, helped organize a daily routine, and favoured contact with other people, which protected her from mental distress:

At that moment our lives changed, and there was more depression, and having the educational courses, the online talks, continuing learning… it was like a bit of liberty, despite being locked. You could see other people’s faces, you could learn, it was a moment to escape from the world around and dedicate to something (…) the best was to build a routine and within this routine to follow the classes, the programs, that helped. (Laura, Focus group)

#### b) Impact on education: continuing learning despite the distance

The Roma was one of the collectives most affected by the disruption of education, due to the lower levels of digital literacy and scarce access to the internet in their households. In this context, the IPRCG placed huge efforts to bridge the digital gap so that every family had access to the virtual environment, and everyone could continue with their courses, leaving no one behind. In this regard, Luis explained that the enhanced connectivity options facilitated by the IPRCG allowed him to both work and study from home and his children to study: “The fact that I could work and study from home, thanks to the resources placed by the IPRCG, allowed my children to keep up with their schoolwork and in close contact with their classmates” (Luis, Interview).

Along with ensuring access to the internet, the IPRCG guaranteed a follow-up mechanism that included phone calls, messages, and video calls on almost a daily basis. In addition, the quality of the educational content was not affected by the shift to virtual spaces. On the opposite, the IPRCG actions were based on the premise that education is one of the most important tools for overcoming poverty and exclusion, and especially in such times of uncertainty, it was crucial to never lower the level or the expectancies towards anyone. In Robert’s words:

It was wonderful. We did the classes online, the teachers were deeply committed, and they did an exhaustive follow-up on us sending homework and making the classes very participative. They showed a huge amount of patience, explaining to us countless times the most difficult concepts until we all understood. (Robert, Interview)

Participants agreed on the key role the teachers and volunteers from the IPRCG played in the success of this action. According to Daniel’s interview, they encouraged Roma families to not give up on their education and to support their children’s performance. Lukas also defended in his interview that the support was so unconditional that he felt that every time he needed any kind of assistance, they were there, ready to help. This comprehensive atmosphere enhanced the sense of belonging of the students through particularly inclusive and engaging classes.

#### c) Impact on employment: better labour conditions during the pandemic

The IPRCG is identified by the participants as a key driver in improving their labour situation during confinement. The strict measures put in place to stop the spread of the virus caused the halt of every non-essential job, displacing many Roma families to a difficult situation. However, those who were able to continue working from home thrived during the hard period of the lockdown. This is the case of Sylvia, who was able to telework thanks to the instrumental support of the IPRCG that gave her a laptop: “They gave me a laptop so that I could work from home and attend online classes, courses, training sessions and online meetings during the entire lockdown” (Sylvia, Interview).

There is consensus among the participants that their participation in the IPRCG activities, especially in those related to education, improved their employment situation. Following with Victor’s experience, he acknowledged that it was the high-quality education he had received through the IPRCG that allowed him to get a better job even during the pandemic, which had an overall positive impact on his life and his family’s. Laura also reflected on this regard:

I continued working the same as a mediator, from home or on-site, I never stopped working because of my education. However, my uncle could not work in the market, because people did not go. Having an education gave us job stability and a different labour prospect. (Laura, Focus group)

Lukas also acknowledged the impact on employment that the IPRCG has had in his life. He experienced the potential of taking part in high-quality and meaningful training courses and perceives a strong link between the education attained and his job opportunities. In his own words:

I think that it is thanks to the training I have completed through the IPRCG that my job opportunities have widened, making a huge difference in my life. I have now better living conditions in all aspects, and it is thanks to IPRCG. (Lukas, Interview)

#### d) Other impacts: bridging access to public aid and instrumental support

The IPRCG was a key resource during the pandemic to facilitate information and access to public aid and related administrative processes. This was especially important because all the administrative steps had to be done online and this was a barrier for many Roma. Having support with these procedures made it possible for many Roma to have access to public aid, as Michael explained:

I think that it is probably one of the best actions that the IPRCG could do because the digital divide was so huge… the fact that people learn to handle these issues by themselves... If the IPRCG had not implemented this action maybe many people would not be able to have such aids. (Michael, Focus group)

Furthermore, the support in handling online administrative procedures helped the participants in the IPRCG to gain digital abilities. According to the participants, many people have become more autonomous with the use of technological tools, which has reduced the digital divide. Victor, who was very active during the confinement helping other Roma with online administrative issues, shared this reflection:

The beginning of the pandemic meant an introduction to the telematic issues, and afterwards, it has evolved a lot, as people could learn thanks to the need of that moment… I think it has been worthwhile for the Roma to make the leap… Today I see those people and they have learnt a lot. (Victor, Focus group)

## Discussion

This study provides evidence on actions that have contributed to preserve the health status of a disadvantaged community in a time of special risk and prevent the increase of already existing inequalities. The conclusions obtained can also be applicable and relevant beyond the present study and be transferred to protect oppressed peoples in other geographies and cultural contexts.

The contrast between the data collected at the beginning of the pandemic from a broad sample of Roma and the evidence gathered afterwards from a sample of participants in the actions of the IPRCG reveals that the IPRCG had a protective effect on the Roma community during the COVID-19 confinement, that counteracted the risk of worsening the situation of inequality that this community traditionally faces and protected their quality of life and well-being.

First, the IPRCG facilitated health literacy that enabled evidence-based decision-making in their everyday life to protect themselves and other people close to them. While the infection rate of the Roma at the beginning of the confinement was high according to the questionnaire data, the dissemination of evidence-based information on health protection helped the participants to avoid infection and challenged the prejudice that blamed them to contribute to the spread of the disease. Previous studies have highlighted the importance of health literacy to improve the health conditions of people of cultural minorities [36], with low educational levels [37, 38] and other vulnerable populations [25–27], as well as its protective factor during the COVID-19 pandemic [29, 30]. Furthermore, in our study, the protective effect covered both physical and psychological health, which is important as the COVID-19 pandemic has had a global impact in both domains [39] and showed racial and ethnic disparities [9, 40].

Secondly, high-quality online education has been crucial to maintain learning opportunities during confinement. This is particularly relevant when it comes to students from vulnerable groups [41], since those spaces allowed them to widening their learning processes while in a safe and supportive online environment, which literature shows as crucial to ensure well-being [42]. The IPRCG facilitated the ability to continue studying online for both children and adults who were involved in training. For this purpose, the educational activities of the IPRCG were transferred to the online modality to ensure education was not interrupted, and informatic equipment was facilitated to those who needed it to keep connected; this was especially important in households with children who had to follow school classes—which was most of the households according to our sample—as it alleviated one of the challenges families faced during confinement.

Thirdly, the IPRCG facilitated the labour inclusion of the participants during the pandemic. More than half of the Roma were unemployed at the beginning of the confinement, according to the questionnaire, and less than 19% could work because they worked in an essential sector or could telework. Data shows that COVID-19 confinement especially affected the most vulnerable and disadvantaged workers, while most professional, scientific, and technical activities that could be transferred to remote service provision were less affected, normally characterized by better employment conditions, and performed by highly skilled workers [43]. For the participants in our study, having been engaged in educational courses of the IPRCG has meant the opportunity to improve their labour conditions and to have more qualified jobs that included the possibility of working from home, which gave economic stability during the confinement.

Fourthly, low-income families and ethnic minorities are identified as vulnerable groups regarding digital inclusion [44]. Although most of the Roma in our sample knew of at least some of the aids that were created to help the population during the COVID-19 pandemic, many of them had insufficient digital competence to deal with the online procedures. The IPRCG provided instrumental support which was always available to facilitate these formalities. Importantly, this support not only helped solve the immediate need to apply for the aids but also contributed to improving the participants’ digital skills.

Finally, as a transversal issue, we identify as a key component of the success of the IPRCG actions the active role of the Roma community itself within the IPRCG. The IPRCG and the actions within it were designed and implemented not only *for* the Roma, but also *with* Roma men and women who participated—within the IPRCG or as volunteers—in disseminating evidence-based information on health promotion, dynamized educational activities and courses, and gave support to access to public aid. Other studies have emphasized the importance of participative and dialogic public policies for the Roma people to reverse their situation of inequality and promote their social inclusion [45]. Their participation in the IPRCG was crucial to know better the challenges that the Roma were facing during the COVID-19 confinement and how these could be addressed successfully.

Our study has focused on the Catalan (native) Roma population. As in any other country and region where the Roma live, there are also migrant Roma—as occurs with non-Roma people. The conclusions of our study could be taken into account to promote the inclusion of the migrant Roma population as well, considering additional actions to facilitate access to the country language. Roma people from other European countries have in the Catalan context a deeper situation of inequality than the Spanish population, created by intersectional or multiple discrimination, and much has still to be done to reduce the inequalities of migrant or refugee Roma in all educational levels and social areas (e.g. access to health, housing, and social participation) [46].

The implications of our findings can extend also beyond the Catalan context. The same as occurs in Spain, the Roma are full-fledged citizens in other European countries, where they nevertheless suffer inequalities and discrimination as well. Therefore, the transformative results obtained in this research could serve to improve the situation of the Roma in other contexts. In this regard, key issues reported in our findings were highlighted in the European Commission’s EUvsVirus Hackathon.^4^ The project *EURomaCommunityChannel* [47], framed in the H2020 ALLINTERACT project (led by Ramon Flecha)^5^, was aimed at responding to the challenge that social distancing and the transition to online entailed for the Roma when facing the COVID-19 pandemic and its consequences in the fields of health, education, housing, and employment. An online platform was created to connect the Roma community in Europe and share relevant information and resources, strengthen the sense of community, facilitate the exchange of rigorous information, and introduce the use of technology, thus contributing to overcoming the digital gap.

Similarly, other cultural minorities, migrant communities, and underprivileged groups affected by inequalities and discrimination could benefit from actions such as those reported here. Data shows that COVID-19 had an unequal impact on the population across countries, being people with low education levels, migrants, racial and ethnic minorities, and low-wage workers those at a higher risk of infection and those who suffered larger losses in employment and income [48]. Facilitating health literacy and digital inclusion to ensure access to information, and protecting vulnerable communities in key areas, such as education and employment, where they face greater discrimination and exclusion and are more likely to be affected in future pandemics, can therefore be crucial to prevent racial and ethnic health and social disparities in multiple contexts, especially if these actions are designed and developed counting with the communities themselves.

Thus, we suggest that the lessons learned from the IPRCG in Catalonia could be transferred to other contexts across countries and guide decision making in different fields (e.g. healthcare, education, social work, public administration) to promote the social inclusion and quality of life of the Roma and other oppressed peoples worldwide, protecting them during future situations of crisis and uncertainty.

## Data Availability

The data that support the findings of this study are available from the corresponding author upon reasonable request.

## Footnotes

1 This is an estimation of the IPRCG, based on the latest data and estimations available and the average population growth. https://dretssocials.gencat.cat/ca/ambits_tematics/accio_comunitaria_i_voluntariat/accio_comunitaria/poble-gitano/pla-integral/2017-2020/

2 The data on confirmed cases of COVID-19 was obtained from *Portal de dades obertes de la Generalitat* (Government of Catalonia): https://analisi.transparenciacatalunya.cat/en/d/c7sd-zy9j/visualization (accessed 1/03/2022). The percentage of cases over the total population was calculated based on a total population of Catalonia on 1/01/2020 of 7.739.758 residents, obtained from IDESCAT: https://www.idescat.cat/pub/?id=aec&n=245&lang=es (accessed 1/03/2022).

3 In Spanish, “expediente temporal de regulación de empleo” (ERTE). It refers to a temporal authorization for companies to suspend an employment contract for a limited period of time.

4 EUvsVirus Hackathon to develop innovative solutions and overcome coronavirus-related challenges. https://www.euvsvirus.org/

5 For more information, see https://allinteract.eu/
